# Saccharibacteria deploy two distinct type IV pili, driving episymbiosis, host competition, and twitching motility

**DOI:** 10.1093/ismejo/wraf119

**Published:** 2025-06-09

**Authors:** Alex S Grossman, Lei Lei, Jack M Botting, Jett Liu, Nusrat Nahar, Jun Liu, Jeffrey S McLean, Xuesong He, Batbileg Bor

**Affiliations:** Department of Microbiology, ADA Forsyth Institute, Somerville, MA 02143, United States; Department of Microbiology, ADA Forsyth Institute, Somerville, MA 02143, United States; West China Hospital of Stomatology, Sichuan University, Chengdu, Sichuan 610093, China; Department of Microbial Pathogenesis, Yale School of Medicine, New Haven, CT 06536, United States; New Haven Microbial Sciences Institute, Yale University, West Haven, CT 06516, United States; Department of Microbiology, ADA Forsyth Institute, Somerville, MA 02143, United States; Institute for Medical Engineering and Science and Department of Biological Engineering, Massachusetts Institute of Technology, Cambridge, MA 02139, United States; Department of Microbiology, ADA Forsyth Institute, Somerville, MA 02143, United States; Department of Microbial Pathogenesis, Yale School of Medicine, New Haven, CT 06536, United States; New Haven Microbial Sciences Institute, Yale University, West Haven, CT 06516, United States; Department of Microbiology, University of Washington, Seattle, WA 98109, United States; Department of Periodontics, University of Washington, Seattle, WA 98195, United States; Department of Oral Health Sciences, University of Washington, Seattle, WA 98195, United States; Department of Microbiology, ADA Forsyth Institute, Somerville, MA 02143, United States; Department of Microbiology, ADA Forsyth Institute, Somerville, MA 02143, United States

**Keywords:** type 4 pili, T4P, *Patescibacteria*, CPR, Saccharibacteria, episymbiosis, episymbiont, microbial ecology, parasitism, microbiome

## Abstract

All cultivated *Patescibacteria*, also known as the candidate phyla radiation, are obligate episymbionts residing on other microbes. Despite being ubiquitous in many diverse environments, including mammalian microbiomes, molecular mechanisms of host identification and binding amongst ultrasmall bacterial episymbionts remain largely unknown. Type 4 pili are well conserved in this group and could potentially facilitate these symbiotic interactions. To test this hypothesis, we genetically targeted pili genes in *Saccharibacteria Nanosynbacter lyticus* strain TM7x to assess their essentiality and roles in symbiosis. Our results revealed that *N. lyticus* assembles two distinct type 4 pili: a nonessential thin pilus that has the smallest diameter of any type 4 pili and contributes to host-binding and episymbiont growth; and an essential thick pilus involved in twitching motility. To understand the role of these pili *in vivo* we developed *Saccharibacteria* competition assays and species-specific Fluorescence in situ hybridization probes. Competition between different *Saccharibacteria* within mock communities demonstrated consistent competitive outcomes that were not driven by priority effects but were dependent on the thin pilus. Collectively, our findings demonstrate that *Saccharibacteria* encode unique extracellular pili that enable their underexplored episymbiotic lifestyle and competitive fitness within a community.

## Introduction

*Patescibacteria*, also known as the candidate phyla radiation or CPR, have long been considered part of the “microbial dark matter” due to their ubiquity in metagenomic and amplicon datasets, and the difficulty of stably culturing strains [1–3]. The many diverse classes of *Patescibacteria* [4, 5] represent a monophyletic lineage hypothesized to encompass 25% [6, 7] to 50% [8] of total bacterial diversity [9]. These ultrasmall bacteria feature highly reduced genomes with limited biosynthetic capacity typical of obligate symbionts [4, 10]. Because most are predicted, or shown, to be dependent on other microorganisms, they cannot be isolated without a host organism. Experimental investigations into *Patescibacteria* have used co-isolation or enrichment methods to identify growth on the surfaces of bacteria [11], surfaces of archaea [12], or cytoplasmically within a paramecium [13]. All *in vitro* cultivation experiments have focused on *Patescibacteria* associated with bacterial host cells, specifically *Saccharibacteria* (historically known as TM7) that grow as epibionts on the surface of *Actinomycetales* hosts [14–16]. *Saccharibacteria* occupy many ecosystems including groundwater [4], soil [17], invertebrate microbiomes [16, 18], vertebrate gut microbiomes [19], and vertebrate oral microbiomes [20]. However, they are most well studied within the human microbiome due to their prevalence in the oral cavity, gastrointestinal tract, respiratory tract, skin, urinary tract, and even breast milk [21].

When growing episymbiotically, *Saccharibacteria* can profoundly alter the lifestyle and phenotype of their bacterial hosts. They can function as parasites, induce changes in morphology and metabolism, and even provide advantages to a host population by protecting against bacteriophages [15, 22–25]. When not attached to a host, *Saccharibacteria* are seemingly incapable of replication; however, they are not metabolically inert. Studies have shown that catabolic pathways, like arginine deiminase, allow planktonic cells to maintain membrane integrity and infectivity during transitional life stages [26]. Despite the ubiquity of *Saccharibacteria* and their ability to alter host physiology, little is known about the molecular mechanisms underlying their metabolism and lifestyle. This dearth of information stems from their evolutionary divergence from non-*Patescibacteria*, difficulties associated with culturing, and, until recently, the lack of genetic tools [27].

*Saccharibacteria* encode multiple predicted surface proteins and structures capable of interacting with host surfaces; however, type 4 pili (henceforth T4P) are strong candidates for host-binding because they are nearly universal across the phylum/class [28]. T4P are complex polymeric filaments composed of pilin monomers. Multiple classes of T4P have been identified (T4aP, T4bP, Tad, and archaeal systems) that are typically distinguished by their signal peptides and their assembly machinery proteins [29]. In T4aP, structural pilins, called major pilins, make up the bulk of each filament and less numerous minor pilins play diverse molecular roles [29]; however, few studies have attempted to investigate *Saccharibacteria* T4P. Previous investigation of *Saccharibacteria* strain TM7i using a nonspecific chemical inhibitor of PilB revealed that T4P were required for twitching motility [16] and a whole genome Tn-seq analysis of *Southlakia epibionticum* revealed that T4P genes were amongst those essential for survival [27]. We used *Nanosynbacter lyticus* strain TM7x and its cognate host *Schaalia odontolytica* strain XH001 as a model system [11] to demonstrate that *N. lyticus* produces two visually differentiable pili with distinct *in vivo* functions (T4P-1 and T4P-2), genetically characterizes the pilins and piliation pathway for T4P-2, and experimentally demonstrates T4P driving host-binding. To provide ecological context, we investigated *Saccharibacteria* competition for host niches, demonstrated co-infection of host cells by multiple *Saccharibacterial* strains, and explored the importance of T4P-2-dependent host tethering on competitive fitness. Our bioinformatic analysis of T4P in *Saccharibacteria*, and the broader *Patescibacteria*, provides a nomenclature and phenotypic foundation for future description of T4P pili in episymbionts.

## Materials and Methods

### Bacteriological culture conditions

All cultures were grown in brain–heart infusion medium (BHI) or BHI supplemented with 5.0% sheep’s blood at 37°C. All BHIs were incubated anoxically for at least 24 h to remove dissolved oxygen and reduce oxidative stress. Cultures were grown in a Whitley A35 (Bingley, UK) with microaerophilic gas mixture (2.0% O_2_, 5.0% CO_2_, 93.0% N_2_) except during anoxic TM7x transformation using a Coy labs (Grass Lake, MI, USA) chamber (2.5% H_2_, 10.0% CO_2_, 87.5% N_2_). Bacterial cultures stored at −80°C in 15.0% glycerol. For competition experiments, strain-specific primers (Table S1) were designed to quantify TM7x, BB004, and TM7-008. For host-binding, competence, and motility experiments, *Saccharibacteria* were quantified using a Malvern Panalytical Nanosight pro (Westborough, MA, USA) standard curve. The bacterial strain list is present in Supplemental Table S2.

### Bioinformatic analyses

*Nanosynbacter lyticus* strain TM7x coding sequences (GCF_000803625.1) were Basic Local Alignment Search Tool (BLAST)-searched using T4aP, T4bP, Com, and Tad systems identified previously [30], analyzed with TXSScan-1.1.0 [31] (E-value cutoff 1*10^−5^), and downloaded as PDB files from the AlphaFold2 database [32, 33] to search for T4P genes. Genes were either assigned the name of a close homolog or assigned an arbitrary letter designation if no homologs could be identified (Table S3). Transcriptomic reads/million data for all *pil* genes were extracted from the Gene Expression Omnibus (GEO) Series: GSE196744 [24].

A phylogeny of select *Saccharibacteria* was generated as described previously [34] with the following modifications. Homologous gene sets were generated using GTDB-Tk v2.4.0 [35] (104 sets total). Maximum likelihood analysis was performed via IQtree (-st AA -m TEST -bb 1000 -alrt 1000) [36] with a general matrix substitution model [37] with empirical frequencies and four gamma rates, then visualized via Dendroscope v3.8.10 [38]. Nodes with <60.0% bootstrap support were collapsed.

Where identified, syntenic loci were visualized in Geneious Prime 2024.0.7 (Boston, MA, USA). SignalP v6.0 [39] was used to predict T4P pilin signal peptides. The GlycoPP v2.0 webserver was used to predict O-linked glycosylation using a CPP + SS model with an increased stringency Support Vector Machine (SVM) threshold of 0.2 [40, 41]. Genomes across *Patescibacteria* were downloaded from the National Center for Biotechnology Information database (NCBI) or the Genome Taxonomy Database (GTDB) (*N* = 868 as of January 2024). Protein sequences for the coding genes in these genomes were queried using the 24 *pil* genes encoded by TM7x. Percent identity and query coverage were averaged at phylum (NCBI-derived sequences) or class (GTDB-derived sequences) levels.

### *N. lyticus* genetics

To generate gene knockout constructs for *N. lyticus*, hygromycin B resistance cassettes were assembled as described previously [27]. Briefly, 300–500 bp homology arms up- and downstream of mutation sites were assembled via NEB (Ipswich, MA, USA) HiFi assembly (E2621) flanking a codon-optimized hygromycin B resistance gene and the *tuf* promoter sequence from *S. epibionticum*. A neutral mutant was generated via insertion into the noncoding region following TM7x_01290. To transform TM7x, cocultures were grown overnight anoxically, mixed with 1 μg of linear DNA construct (into 500 μl) and incubated for 6 h. After incubation, 3 ml of BHI containing dilute *S. odontolytica* (OD_600_ = 0.05) and hygromycin B (final concentration 150 μg/ml) was added to transformation mixtures. All cultures were incubated anoxically for 24 h and then passaged 1:10 into *S. odontolytica* (OD_600_ = 0.05) with hygromycin B (150 μg/ml) four times. Passage five and later were grown microaerophilically, without hygromycin B to enhance outgrowth of transformants, because growth in hygromycin B is quite slow. Our definition/metric for outgrowth is fold change in TM7x at passage 6 relative to passage 4. When NS1 transformation cultures reached >70% *N. lyticus* saturation, transformants were plated for isolation. A Ken-A-Vision T-22021 stereomicroscope (Kansas City, MO, USA) was used to identify “irregular” colonies based on their inconsistent morphology as previously described [42], which were then screened via polymerase chain reaction (PCR) amplification (≈6.3% of colonies transformed). All mutants confirmed via Oxford Nanopore whole genome sequencing at Plasmidsaurus (Eugene, OR, USA). All primers are described in Supplemental Table S1.

### Cryogenic-electron tomography

For imaging of pili, Cryo-ET samples were prepared using copper grids with holey carbon support film (300 mesh, R2/2, Quantifoil) as previously described [43]. The grids were glow-discharged before depositing 5 μl of TM7x/host cell solution in phosphate buffered saline (PBS) (OD_600_ ≈ 1.25) with 10 nm gold fiducial beads. Grids were blotted with Whatman filter paper and frozen in a cryogenic mixture of 37% liquid ethane and 63% liquid propane cooled with liquid nitrogen using a homemade gravity-driven plunger apparatus or a Leica Microsystems GP2 plunger set to 25° and 95% relative humidity (Wetzlar, Germany).

The plunge-frozen grids were clipped into AutoGrids, mounted into the autoloader under liquid nitrogen, and docked onto a 300 kV Titan Krios electron microscope (Thermo Fisher Scientific, Waltham, MA, USA) equipped with a K3 BioQuantum direct electron detector (Gatan), a Volta Phase Plate (VPP), and a BioQuantum energy filter (Gatan). SerialEM [44] was used to collect single-axis tilt series around −5 μm defocus with VPP, with a cumulative dose of ≈60 e^−^/Å covering tilt angles from −48° to 48° (3° increment). Images were acquired in a dose-symmetric scheme using a FastTomo script with an effective pixel size of 2.148 Å at the specimen level. Recorded images were drift corrected by MotionCor2 [45] and stacked by IMOD [44, 46]. Tilt series were aligned by IMOD with the patching tracking method. Tomograms were reconstructed in IMOD using the Simultaneous Iterative Reconstruction Technique algorithm. Pili were manually detected, segmented, and measured using Region of Interest and Ruler tools in Dragonfly version 2022.2 (Comet Technologies Inc, Shelton, CT, USA). Pili were imaged and quantified for 5–10 cells per genotype.

### *S. odontolytica* binding assay

Prior to inoculation of host cultures with episymbionts, *Saccharibacteria* strains had to be isolated as previously described [42]. Three to four biological replicates were performed for each genotype, except wildtype that was replicated with every experiment (*N* = 8). Isolates were normalized by OD_600_ and re-introduced to *S. odontolytica* hosts at a multiplicity of infection (MOI) of 0.1. Cocultures were incubated for 24 h, then the percentage of host cells bound by episymbionts was determined via phase contrast microscopy with Nikon Eclipse E400 (Tokyo, Japan). For every replicate, bound and unbound host cells within 8–10 microscope fields were manually counted, giving a total of 218–1279 cells per treatment (476 average). We define a “bound cell” as a host cell that is in direct physical contact with at least one episymbiont at the time of imaging. For quercetin treatments (100 μM), 1000× stocks were prepared fresh in Dimethyl sulfoxide (DMSO) before use.

### *N. lyticus* pilin mutant growth dynamics

Prior to inoculation of *S. odontolytica* cultures with episymbionts, TM7x strains had to be isolated as described above. Each TM7x genotype was added to *S. odontolytica* at an MOI of 0.1 and then incubated as described above. Every 24 h, OD_600_ (ThermoScientific Genesys30) and phase contrast images (Nikon Eclipse E400) would be acquired prior to cultures being passaged via 1:10 dilution into BHI media.

### *Saccharibacteria* twitching motility

To visualize twitching motility, overnight cocultures of *S. odontolytica* and each TM7x genotype were placed onto ≈350 μm-thick 0.8% agar pads (w/v) and allowed to dry for 5 min. Agar pads were inverted onto MatTek glass-bottom petri dishes and phase contrast–imaged under anoxic conditions (Nikon ECLIPSE Ti). Images were taken every 3 s for 3 min. Paths of movement were traced and measured in Fiji [47]. Between 31 and 34 cells were traced for every tested genotype except wildtype (*N* = 58) and Neutral site 1 (*N* = 48). Wildtype *N. lyticus* were also treated with 200 μM quercetin and DMSO vehicle to test T4P (*N* = 20). Finally, cells were heated for 10 min at 97°C as a heat-killed control (*N* = 10).

### *N. lyticus* competence assay

To determine the rate of transformation for TM7x, linear constructs were designed to insert pTuf mNeonGreen into NS1. NEB HiFi assembly master mix and PCR amplification were used to amplify the linear construct with 300 bp homology arms. Anerobic overnight cocultures containing each *N. lyticus* genotype were transformed with 1 μg of NS1::mNeonGreen construct and incubated for 6 h. No DNA control and lysed cell controls were performed using wildtype *N. lyticus*. After incubation, dilute *S. odontolytica* (OD_600_ = 0.05 in BHI) was added for outgrowth. After 24 h of anaerobic growth, cells were rinsed 2× in PBS. The lysed cell control was heated to 97°C for 10 min, frozen, and heated again for 5 min. Extracellular DNA was removed via 30-min NEB DNase I (M0303) treatment. gDNA was extracted using MasterPure Gram-Positive DNA Purification kits (MGP04100), diluted 1:10, and quantified using TM7x specific primers and mNeonGreen-specific primers. Data were log_10_-transformed for normality. All primers are described in Supplemental Table S1.

### *Saccharibacteria* competition

For tripartite competition, TM7x, BB004, and TM7-008 were introduced into overnight *S. odontolytica* cultures with individual MOIs of 0.1, followed by outgrowth and passaging every 24 h for 12 days. Pairwise competitions started with established cocultures of *S. odontolytica* infected with each experimental *Saccharibacteria*, then separately infected each coculture with the remaining two species (MOI 0.1) and passaged daily for 8–12 days. For mutant TM7x competition, the coculture containing BB004 was infected with each TM7x genotype (MOI 0.1) and passaged for 10 days. Competitions were performed in biological triplicate.

### Fluorescence *in situ* hybridization staining

To visualize co-infecting *Saccharibacteria* strains on host cells, whole-mount fluorescence *in situ hybridization* (FISH) was performed as previously described [48, 49] with adjustments. One millilitre of cocultures was fixed (4.0% formaldehyde) at 4°C for 1 h and digested (2 mg/ml lysozyme) at 37°C for 9 min. Fixed cells were incubated for 2 h in hybridization solution supplemented with appropriate probes (5 ng/μl) at the indicated temperatures (Table S4), taking care to avoid light. Cells were rinsed in wash buffer for 15 min (215 mM NaCl, 20 mM Tris, pH 7.5, 5 mM Ethylenediaminetetraacetic acid), then rinsed twice in 0.1× SSC for 10 min (15 mM NaCl, 150 μM sodium citrate). Cells were imaged using a Zeiss LSM 780 Confocal Microscope. All oligonucleotide probes are described in Supplemental Table S4.

### Quantification and statistical analysis

All statistical analyses were performed in Prism 10.3.0 (GraphPad Software, San Diego, CA, USA). Transcriptomics was analyzed via the Holm–Šídák adjusted unpaired *t*-test. All other statistical comparisons were analyzed via one-way Analysis of Variance (ANOVA) with Dunnett’s multiple comparison test. Differences with a *P*-value <.05 were considered significant.

## Results

### TM7x has multiple T4P loci, encoding two distinct systems

**Figure 1:**
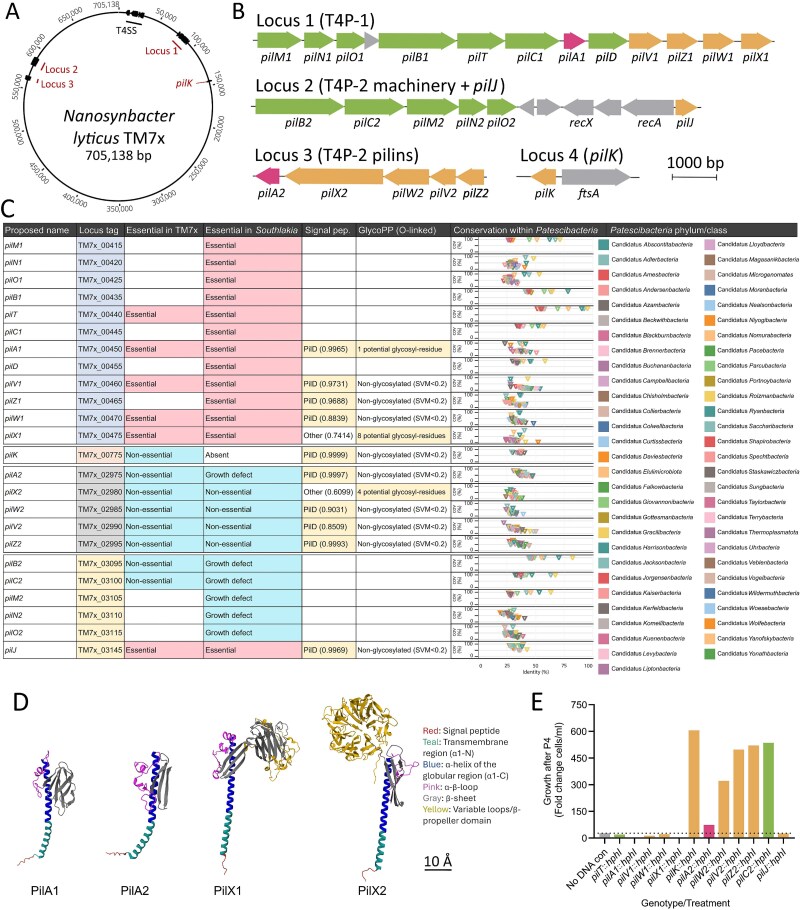
Identification and description of the T4P genes encoded by *N. lyticus*. Bioinformatic identification of T4P genes within *N. lyticus* TM7x (A) indicates four genomic loci (B). These loci encode two distinct T4P extrusion systems (green) and 12 putative pilins (major pilins in pink, minor pilins in yellow). (C) A table summarizes all *pil* genes named in this manuscript, compares their essentiality in *N. lyticus* to previously reported TnSeq results in the related bacterium *S. epibioticum* [27], and indicates bioinformatically predicted modifications (signal peptidase sites and potential for O-linked glycosylation). Conservation of each identified *pil* gene was assessed across a database of genomes representing 56 *Patescibacteria* phyla. Graphs indicate averaged percent coverage (*y* axes) vs percent identity (*x* axis). (D). Structural predictions of major pilins, as well as the most highly expressed minor pilins, reveal archetypical “lollipop” structures common to T4aP systems, featuring a hydrophobic N-terminal α-helix followed by one or more globular domains. (E) Transformation with a selection of allelic exchange cassettes reveals which pilin genes are essential for *N. Lyticus* survival and which are dispensable. Outgrowth rate of mutant bacteria post-transformation (TM7x density after four passages with hygromycin B/TM7x density after two additional passages without hygromycin B) offers insight into potential growth defects (Δ*pilB2* and Δ*pilX2* isolated without a full outgrowth period).

We bioinformatically identified all pili genes within *N. lyticus* TM7x and compared them to previously characterized T4P. A three-pronged approach utilizing a database of diverse T4P systems (T4aP, T4bP, Com, and Tad) [30], TXSScan-1.1.0 [31] analysis, and AlphaFold derived pilin structural predictions resulted in identification of two sets of T4aP machinery genes and 12 pilin genes (Fig. 1A and B, Table S3). Whenever possible, genes were assigned the name of their closest T4P homolog; however, due to the low homology of pilin sequences, many were assigned arbitrary letters. T4P locus one (TM7x_00415–00475) encodes sufficient genes to produce a pilus, including the membrane platform (*pilC1*), assembly ATPase (*pilB1*), retraction ATPase (*pilT*), alignment factors (*pilM1*, *pilN1*, and *pilO1*), Signal peptidase III (*pilD*), and five predicted pilins (Fig. 1A and B). T4P locus two (TM7x_03095–03145) lacks a retraction ATPase but encodes a mostly complete set of assembly machinery genes (*pilC2*, *pilB2*, *pilM2*, *pilN2*, and *pilO2*), a recombinase operon (*recAX*), and a single predicted pilin. T4P locus three (TM7x_02975–02995) encodes five predicted pilins. T4P locus four (TM7x_00775) contains an orphan pilin. Given the presence of redundant assembly machineries, we hypothesized that TM7x produces at least two distinct filaments: T4P-1 from locus one and T4P-2 from locus two. Within TM7x transcriptomic datasets [42], pilin expression accounted for ≈1.8% of TM7x transcripts, and expression of 13 out of 24 T4P genes was significantly elevated after stable episymbiosis (Passage 6) (Fig. S1A). The most highly expressed pilins were *pilA2* and *pilA1*, strongly suggesting that these are major pilin subunits. The most highly expressed putative minor pilins were *pilX2* and *pilX1*, which both encode expanded C-terminal domains.

To assess conservation within *Patescibacteria*, a database of 868 genomes across 56 phyla/classes [34] (excluding *Saccharibacteria*) was assembled and BLAST-analyzed for closest homologs (Fig. 1C, Table S5). Pilus machinery genes (*pilB*, *pilT*, *pilM*, and *pilC*) were well conserved, having homologs with 100% query coverage in most phyla/classes at higher amino acid identities (25%–75% identity). Conversely, pilin genes had low levels of conservation, typically sharing 10%–25% coverage with query sequences, driven predominantly by N-terminal features like signal peptides and transmembrane α-helices. Representative loci from *Saccharibacteria* including close relatives of *N. lyticus* from *Nanosynbacteraceae*, *Saccharimonadaceae*, and GTDB f__UBA1547 (clade G1) and distant relatives from *Nanosyncoccaceae* (G3), *Nanoperiomorbaceae* (G5), and *Nanogingivalaceae* (G6) were compared [34] (Fig. S1B)*.* All clade G1 genomes encoded two T4P assembly machinery sets like TM7x, differentiated by only their complement of minor pilins (Fig. S1C–F). More distant *Saccharibacteria* from outside of clade G1, conserved T4P locus one, however, had no homology to locus two, three, or four, suggesting that these organisms only produce a single T4P filament.

AlphaFold2 [50] prediction of TM7x pilins demonstrated a range of diverse structures in the globular region (Fig. S2A). The putative major pilins PilA1 and PilA2 were predicted to have prepilin peptidase (PilD) signal peptides and to adopt classical “lollypop” structures associated with T4aP pilins [51–53], wherein a globular domain is attached to an N-terminal hydrophobic α-helix (Fig. 1C and D, Fig. S2). Putative minor pilins also have lollipop architecture, though most carry additional features on their globular domains (Fig. S2A and B). Most are elongated by variable loops (PilV1, PilW1, PilV2, PilW2, and PilK), and a few have additional C-terminal domains (PilX1 and PilX2). Specifically, PilX1 has a β-Jelly Roll-like domain and PilX2 has a hepta-bladed β-propeller domain with RCC1 repeats (Fig. 1D). PilX1 and PilX2 were not predicted to have PilD signal peptides, potentially indicating that these pilins are post-translationally modified without PilD or prime pilus polymerization like PilY1 in *Myxococcus* [54] (Fig. 1C). Structural predictions for the extrusion/retraction machinery proteins PilB and PilT were similar to non-*Patescibacteria* T4aP, possessing adenosine triphosphate (ATP) binding and hydrolysis sites [55] (Fig. S2C). Other T4P are decorated with O-linked glycans that impact bacterial motility and virulence [56, 57]. GlycoPP V2.0 predicted three TM7x pilins are potentially O-glycosylated, PilA1, PilX1, and PilX2 [40, 41] (Fig. 1C).

### Generation of T4P pilin knockout mutants

We attempted to delete pilin genes from all four genomic loci, as well as extrusion ATPase *pilB2* and membrane platform *pilC2* via allelic exchange [27]. These genes were targeted to separately interrogate the importance of each T4P locus and to disentangle the roles played by T4P assembly machinery, major pilins, and individual minor pilins. A neutral site mutant was generated (NS1:*hphI*) as a technical control. All targeted pilins in locus one (*pilA1*, *pilV1*, and *pilW1*) and two (*pilJ*) could not be deleted, suggesting that they are essential for TM7x survival. All pilins in locus three (*pilA2*, *pilX2*, *pilW2*, *pilV2*, and *pilZ2*) and four (*pilK*) were nonessential (Fig. 1E). The rate of TM7x population expansion postselection was reduced in TM7xΔ*pilA2.* Gene essentiality within TM7x was in agreement with the essential genes detected in the related *Saccharibacteria S. epibionticum* [27] via Tn-seq mutagenesis, including the growth defect introduced by mutating *pilA2*, indicating that T4P play similar roles within the families *Nanosynbacteraceae* and *Saccharimonadaceae*.

### Two distinct pili filaments are assembled on the surface of TM7x

**Figure 2:**
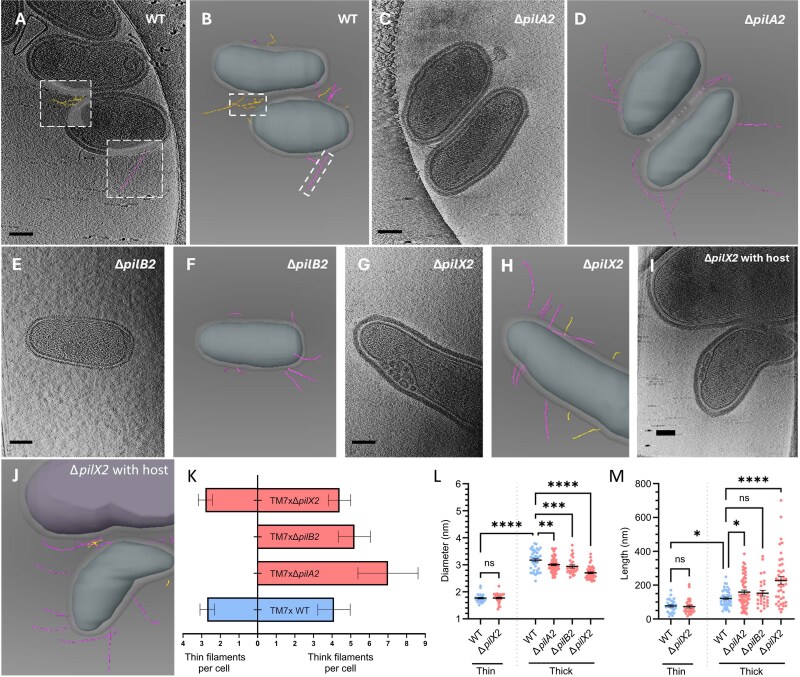
Cryo-ET reveals two distinct T4P filaments, distinguishable by diameter. Electron tomography of planktonic TM7x (*N* = 10) (A) and 3D reconstruction of surface structures (B) reveals two distinct filament types present on the surface, thin pili with diameters of ≈1.8 nm (left inset box) and thick pili with diameters of ≈3.2 nm (right inset box). All scale bars are 100 nm long. TM7xΔ*pilA2* cells (*N* = 8) (C, D) and TM7xΔ*pilB2* cells (*N* = 5) (E, F) have no thin pili on their surface and show modest changes in thick pilus diameter and length (K-M). TM7xΔ*pilX2* (*N* = 10) still have thin filaments present (G, H) but show a larger impact on the diameter and length of the thick filament. No mutant completely removed the ability for TM7x to associate with its host cells, as shown with a representative TM7xΔ*pilX2* (IJ). * = *P*-value ≤.05, ** = *P*-value ≤.01, *** = *P*-value ≤.001, **** = *P*-value ≤.0001.

To validate bioinformatic predictions, cryogenic-electron tomography (henceforth Cryo-ET) was used to observe TM7x surface filaments on free-floating TM7x cells [42]. Tomograms of wildtype TM7x indicated ≈6.5 filaments/cells, with a bimodal distribution of diameters centered around 1.8 nm (2.5/cell) and 3.2 nm (4/cell), suggesting two distinct surface appendages (Fig. 2A  B, K, and  L, Fig. S3A and B), henceforth referred to as thin pili and thick pili. These widths are small for T4P, which typically have diameters between 5 and 8 nm [29]. Thin pili had an average length of 77.4 nm (15–171 nm range), and thick pili had an average length of 121.9 nm (45–249 nm range) (Fig. 2M).

Tomograms of TM7xΔ*pilA2* (Fig. 2C and D, Fig. S3C and D) indicated ≈7 filaments/cell, composed entirely of thick pili (≈3.2 nm diameter) (Fig. 2K and L). The thin filaments are completely removed by deleting *pilA2*, confirming that they are T4P despite their narrow diameter. Thick pili observed on TM7xΔ*pilA2* were 70.7% more numerous and significantly longer (158.9 nm average) than wildtype thick pili (Fig. 2M). Tomograms of TM7xΔ*pilB2* indicated ≈5.2 filaments/cell composed entirely of thick pili, much like TM7xΔ*pilA2* (Fig. 2E, F, and  K–M, Fig. S3E and F). Because loss of *pilB2* removes all thin pili, we conclude that the thin pilus is T4P-2, composed of assembly machinery encoded in locus two and pilins encoded in locus three. We hypothesize that the thick pilus is T4P-1, but we cannot directly connect the two on a genetic level because the essential T4P-1 pilins cannot be deleted. Tomograms of TM7xΔ*pilX2* (Fig. 2G and H, Fig. S3G and H) indicate ≈4.4 thick filaments/cell and ≈2.7 thin filaments/cell (Fig. 2K). TM7xΔ*pilX2* were most like wildtype cells; however, their thick pili had significantly narrower diameters (2.7 nm average) and longer lengths (227.5 nm average) (Fig. 2L and M), suggesting an alteration of pilin subunit composition or conformation. We did not observe pili concentrating in between episymbionts and adjacent hosts; however, electron opacity differences between *S. odontolytica* and *N. lyticus* make it difficult to visualize host cells and TM7x simultaneously, so our analysis focused on unbound cells. (Fig. 2I and J, Fig. S3G).

### T4P-2 contributes to TM7x host-binding but not to motility or competency

**Figure 3:**
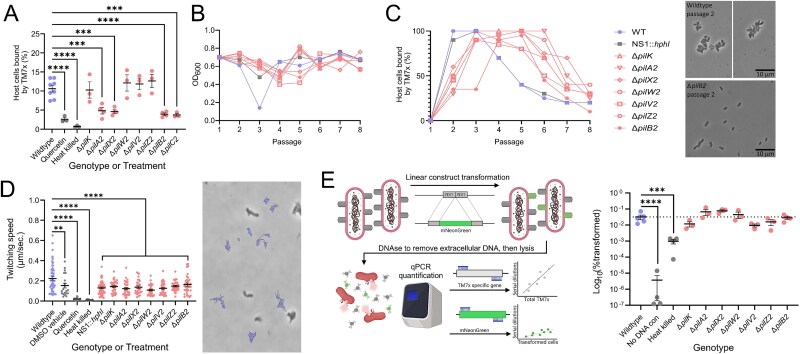
Characterization of host-binding, twitching motility, and genetic competence in TM7x T4P-2 mutants. Exposing *S. odontolytica* cultures to TM7x reveals ≈10.6% of hosts bound by wildtype episymbionts after 24 h, which is reduced by half or more in the *pilA2*, *pilX2*, *pilB2*, and *pilC2* mutants (A). Application of PilB/PilT inhibitor quercetin induces a similar reduction. Repeated passaging of episymbiont-infected cultures reveals modestly reduced rates of host killing (B), host adaptation, and TM7x population growth (C) in pilin mutants, most clearly visible in representative images from passage 2. For host adaptation experiments, individual genotypes were only tested once; however, replication of the adaptation/growth delay in all seven T4P mutants tested reinforces the growth defect induced by even subtle disruption of T4P-2. Timelapse photography (D) of cocultures allowed tracking of TM7x twitching motility and showed that all T4P-2 mutants were motile and quercetin-treated cells were not. A representative section of timelapse microscopy (3 min) demonstrates the stochastic twitching motions of wildtype TM7x. Transforming TM7x cells of each genotype with an mNeonGreen gene (E) demonstrated that all T4P-2 mutants maintained wildtype levels of competence. * = *P*-value ≤.05, ** = *P*-value ≤.01, *** = *P* value ≤.001, **** = *P*-value ≤.0001.

To test the contribution of T4P to episymbiotic host-binding, axenic host bacteria were infected with each TM7x genotype at an MOI of 0.1 (1:10 TM7x:host ratio), incubated for 24 h, and imaged to determine what percentage of hosts had been bound by TM7x. When exposed to wildtype TM7x for 24 h, ≈10.6% of host cells were bound by TM7x (Fig. 3A). Deletion of the minor pilins *pilK*, *pilW2*, *pilV2*, and *pilZ2* had no significant effect on host-binding. However, a significant defect in host-binding resulted from deletion of major pilin *pilA2* (4.8% of host cells bound), minor pilin *pilX2* (4.6%), ATPase *pilB2* (3.9%), and membrane platform *pilC2* (3.7%) (Fig. 3A). The similarity of the binding defects observed suggests that major pilin PilA2 and minor pilin PilX2 are likely assembled into the same filaments and work in conjunction to interact with host bacteria. This is supported by the Cryo-ET results (Fig. 2) where deletion of *pilA2* prevented assembly of T4P-2 (thin), and deletion of *pilX2* only impacted filament counts and length. Observed binding defects do not completely prevent host-binding, nor do they prevent the mutants from eventually reaching high densities (Fig. 1E).

To determine if pilin mutants have altered growth dynamics, mutants were re-introduced to axenic *S. odontolytica* cultures and passaged daily until populations stabilized. Wildtype TM7x induced host population crash at passage 3 as observed previously [42], the neutral site mutant (NS1::*hphI*) had a smaller host crash at passage 3, and all pilin mutants induced a small host crash at passage 4 (Fig. 3B). The reduced crash phenotype seen in all mutants suggests that the transformation process decreases the amount of stress TM7x applies to hosts. The delayed host die-off seen in T4P-2 mutants relative to the neutral mutant suggests that T4P-2 defects subtly impaired host-binding or growth rate. Wildtype TM7x and the neutral mutant achieved high levels of host-binding by passage 2 (90–100%), most pilin mutants achieved high levels at passage 3, and TM7xΔ*pilB2* was delayed until passage 4 (Fig. 3C). The neutral mutant stabilized its population at passage 6 (20%–30%) and T4P-2 mutants stabilized at passage 7. Collectively, these data suggest that disruption of T4P-2 decreases the host-binding rate, particularly during early time points.

T4P are known to power gliding and twitching motility; thus, all mutants were examined for motility defects [58]. When suspended on agar gel, *Saccharibacteria* can be seen to “twitch” rapidly across the surface (Supplemental Video S1). Wildtype TM7x had an average speed of ≈0.22 μm/second and a max speed of 0.65 μm/second (Fig. 3D). The neutral site mutant (average 0.13 μm/second) and all T4P mutants showed similar twitching defects, suggesting that transformation causes a subtle twitching motility defect, but loss of T4P-2 does not. Because the T4P inhibitor quercetin inhibited twitching (0.02 μm/second), and loss of T4P-2 did not, our data indicate that T4P-1 likely drives twitching motility. Next, we tested if T4P-2 contributes to natural competence in TM7x. Cocultures containing each TM7x genotype were transformed with a green fluorescent protein (GFP) homolog to quantify competency (Fig. 3E). Approximately 0.01%–0.07% of wildtype TM7x were transformed (≈1:3000 cells); lysed and untransformed controls were significantly lower than wildtype. Pilin mutants showed similar competency rates to wildtype (0.01%–0.08%), suggesting that T4P-2 does not contribute to competency (Fig. 3E).

### *Saccharibacteria* species competition is modulated by T4P

**Figure 4:**
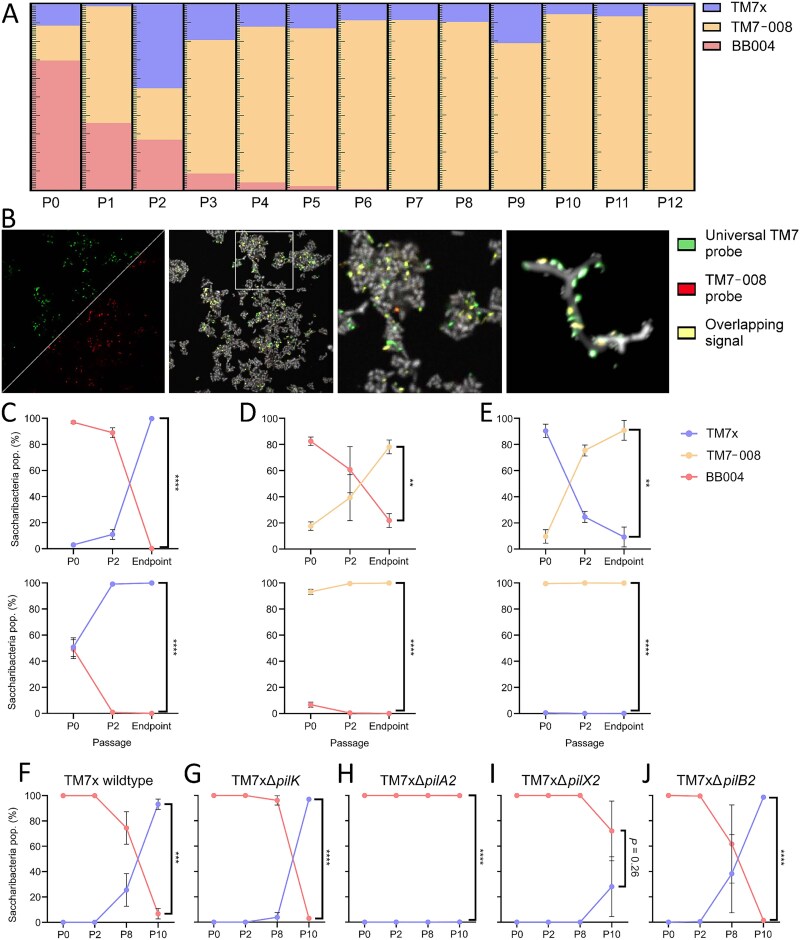
*Saccharibacteria* competition allows for host co-infection, results in consistent trophic successions, and is impacted by T4P-2. Co-infection of *S. odontolytica* cultures with heterospecific *Saccharibacteria* strains (TM7x, TM7-008, and BB004) followed by repeated passaging reveals consistent trophic successions, resulting in TM7-008 dominance (A). FISH imaging of cocultures using a general *Saccharibacteria* probe and strain-specific probe (TM7-008) 8 passages after epibiont addition demonstrated that strains could co-infect a single host (B). Subsequent cell wall staining using conjugated wheat germ agglutinin that preferentially binds recently deposited or exposed cell walls revealed that *Saccharibacteria* strains have distinct effects on host growth and morphology, such as spheroid formation in TM7-008 cocultures (C–F). Pairwise combinations of TM7x/BB004 (C), TM7-008/BB004 (D), and TM7x/TM7-008 (E) reveal more consistent trophic successions, and demonstrate that priority effects may offer a temporal advantage, but do not alter competitive outcomes. By co-infecting bacterial host cells with TM7x T4P mutants and wildtype BB004 (F–J), we demonstrate that the host-association defects induced in TM7xΔ*pilA2* and TM7xΔ*pilX2* significantly impair TM7x’s competitive fitness. * = *P*-value ≤.05, ** = *P*-value ≤.01, *** = *P*-value ≤.001, **** = *P* value ≤ .0001.

Competition would be expected in the oral cavity, which contains hundreds of microbial species, composed of 1%–21% *Saccharibacteria* [14, 59, 60]. There are no prior studies on *Saccharibacteria* competition. To establish an assay, three species of *Nanosynbacteraceae* (TM7x, BB004, and TM7-008) were combined to create tripartite and pairwise competitions. In tripartite competition, all species were added to a host culture, passaged daily, and enumerated via qPCR amplification (Table S1). TM7x consistently competed poorly during passage 1, had a growth spike at passage 2, and then persisted around 1*10^4^ cells/ml (Fig. 4A, Fig. S4A and B). BB004 steadily declined each passage until approaching extinction (≈100–10 cells/ml). TM7-008 rapidly increased in abundance during the first few passages, then remained the dominant episymbiont.

To observe competition directly, we developed strain-specific FISH probes for 16S rRNA. To assess probe specificity, cultures containing each *Saccharibacteria* were multiplex-stained with a universal *Saccharibacteria* probe (TM7-567) [61] and potential strain-specific probes (Fig. S5A and B), then colocalization was quantified. High specificity probes were identified for TM7x (TM7x-3) and TM7-008 (TM7-008-2) (Table S4). Probes were designed for BB004, but none were sufficiently specific. TM7x vs TM7-008 competitions and tripartite competitions were multiplex stained at passages 2 and 8 (Fig. 4B, Fig. S6). At passage 2, *Saccharibacteria* existed diffusely throughout the cultures. When visualized again at passage 8, the density of *Saccharibacteria* had decreased, reflecting host adaptation. When looking at single *S. odontolytica* cells, we can see TM7x and TM7-008 cells growing in close physical proximity on the same host (Fig. 4B), showing no direct signs of spatial exclusion or neighbour killing.

We combined strains in a pairwise manner to determine competitive hierarchy. To determine if an ecological succession of *Saccharibacteria* species was possible, we introduced competitors into established episymbiotic cocultures. When adding TM7x to established BB004, TM7x achieved dominance after passage 2. However, when BB004 was added to established TM7x, TM7x became dominant before passage 2 despite starting passage 0 with a nearly equivalent concentration of BB004, seemingly due to a low starting population at time of inoculation (Fig. 4C, Fig. S4C and D). Having an established coculture provided temporal advantage but did not affect the outcome. When competing BB004 and TM7-008, a similar pattern emerged where TM7-008 always became dominant, but taking over an established co-culture took longer (Fig. 4D, Fig. S4E and F). When competing TM7x and TM7-008, TM7-008 always became dominant and managed to do so by passage 2 (Fig. 4E, Fig. S4G and H). In summary, TM7-008 outcompeted both competitors andTM7x only outcompeted BB004, illustrating that priority effects did not impact ecological succession.

TM7x’s ability to outcompete BB004 was examined using TM7xΔ*pilK*, TM7xΔ*pilA2*, TM7xΔ*pilX2,* and TM7xΔ*pilB2* mutants. When inoculated into established BB004 co-cultures, TM7x, TM7xΔ*pilK*, and TM7xΔ*pilB2* became the predominant *Saccharibacteria* between passage 8 and 10 (Fig. 4F, G, and  J, Fig. S4I, J, and  L). Conversely, TM7xΔ*pilA2* and TM7xΔ*pilX2* did not manage to outcompete BB004 (Fig. 4H and I). Absolute abundances indicate that both mutants struggled to become established and did not start growing until after passage 8 (Fig. S4K and M). Like our growth dynamic assays (Fig. 3A and B); this suggests that PilA2 and PilX2 mediate early bacterial host interactions important for *Saccharibacteria* competition but are not essential for long-term binding.

## Discussion

**Figure 5:**
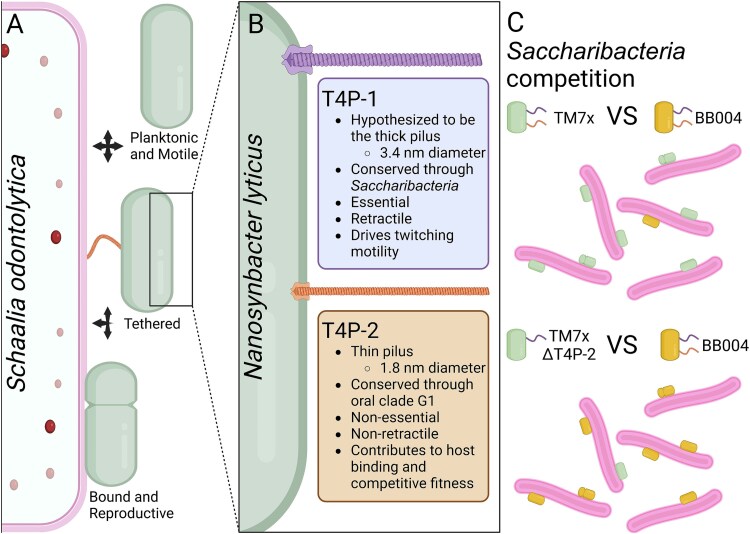
Conceptual model of T4P function in *N. lyticus*. (A) To propagate between hosts, *Saccharibacteria* like *N. lyticus* need to alternate between a planktonic, motile life stage and a host-bound reproductive stage. Within our model, host-binding involves multiple steps and long-range host tethering through T4P-2 is one of the initial steps. (B) Microscopy and phenotypic profiling of T4P mutants revealed that *N. lyticus*, and likely all G1 clade *Saccharibacteria*, produce two distinct pili; a 3.4 nm pilus that we hypothesize to be the retractile T4P-1 and a 1.8 nm pilus that we demonstrated to be T4P-2. (C) When T4P-2 was removed from cells, host-binding was delayed significantly and *N. lyticus* TM7x lost its competitive advantage over another *Saccharibacterium*, BB004. Created in BioRender. Bor, B. (2025) https://BioRender.com/n44o152.

Our investigation of T4P in *Saccharibacteria* reveals highly specialized macromolecular filaments that contribute to cellular functions and host association. *Nanosynbacter lyticus* encodes two distinct T4P (T4P-1 and T4P-2), indicating a potential division of labor for extracellular functions like twitching and host detection. The full extent of T4P-1’s functional roles cannot be determined at this time due to their essentiality for cell survival. Similarly, we cannot confirm that the thick pilus identified in CryoET images is T4P-1 directly. However, *N. lyticus* is only predicted to encode three filaments based on its genome, two T4P and one Type 4 Secretion System (T4SS). Using process of elimination, the thick pilus is unlikely to be a T4SS based on its flexibility and small size, being less than half the size of the smallest T4SS and barely larger than the diameter of double-stranded DNA (~2 nm) [62]. Future studies could attempt to definitively link the thick pilus and T4P-1 using ImmunoEM with PilA1-specific antibodies. T4P-2 significantly increases the rate of host-binding and the competitive fitness of the epibiont. Based on this observation, we propose a multiphase binding process wherein T4P-2 acts as an initial, long-range host recognition factor by binding a host receptor, tethering *N. lyticus* (Fig. 5A and B). Subsequent twitching motility through T4P-1 can bring tethered cells into physical contact so that additional close-proximity adhesins can establish a direct bond, as previously proposed [27]. Except for PilX2, no single minor pilin was required for T4P-2 function. PilX2’s C-terminal β-propeller, noncanonical signal peptide, and potential for glycosylation make it an excellent candidate for the adhesin component of T4P-2, similar to PilY1 from *P. aeruginosa*, which binds integrin glycoproteins and also has a hepta-bladed β-propeller [63]. Finally, we demonstrate that host cell tethering via T4P-2 is required for *N. lyticus* to effectively compete with heterospecific *Saccharibacteria*, highlighting the importance of rapid and long-range host attachment to episymbiont success (Fig. 5C).

The T4P produced by *N. lyticus* reflect their unique episymbiotic lifestyle. Although bacteria such as *T. thermophilus* [64] and *Vibrio parahaemolyticus* [65] produce two distinct T4P filaments, it is not common. Expression of two metabolically expensive piliation systems throughout all environmental and mammalian-associated clade G1 *Saccharibacteria* (*Nanosynbacteraceae* and *Saccharimonadaceae*) suggests that they provide significant fitness benefits. The presence of two distinct systems indicates either a division of expression timing (not supported by cryoET or transcriptomic data) or a division of otherwise incompatible functions. One division of labour suggested by our data could be retraction. T4P-1 encodes a retraction ATPase and appears to drive twitching motility. Conversely, T4P-2 does not appear to encode a retraction ATPase and was not essential for twitching motility. Hypothetically, T4P-1 could be responsible for retraction-dependent processes whereas T4P-2 acts as a passive mediator of surface interactions within the oral microbiome TM7x was isolated from.

Another distinction between these T4P and canonical T4P is their size. With an average diameter of 1.8 nm, TM7x T4P-2 appears to be the thinnest T4P discovered. T4P-1 (3.2 nm diameter) is also thinner than average, particularly for a force-generating pilus implicated in twitching motility. For example, 6–7.5 nm T4P from *M. xanthus* apply up to 149 pN of force [66, 67] and ≈6 nm T4P from *Neisseria gonorrhoeae* [68, 69] can apply 110 pN. Future studies could aim to measure the tensile strength of these filaments to determine if they have evolved greater relative strength to compensate for reduced width. Conversely, reduced pilus widths could be an adaptation for reducing metabolic costs for filaments that do not need to withstand large forces. If genuinely non-retractile, T4P-2 may be more functionally analogous to thin curli fibers [70] than to other T4P, highlighting the versatility of these ancient structures.

The dearth of previous studies examining T4P in *Patescibacteria* means that many open questions surround assembly machinery superstructure, extrusion/retraction regulation, filament polymerization, and minor pilin integration. No single study could answer all these disparate questions, but our findings provide a foundation to elaborate upon. Techniques and discoveries from our studies will facilitate molecular characterization of T4P in *Saccharibacteria* and throughout *Patescibacteria*. T4P are versatile molecular machines that have evolved to fill many roles in distinct lineages, and our findings expand our knowledge of this evolutionary flexibility. Furthermore, the lessons we learn about *Patescibacteria* host-binding factors and competition dynamics may, in turn, enable prediction of episymbiont host ranges or *in vitro* culture conditions for organisms beyond *Saccharibacteria*.

## Supplementary Material

MergedSupplementalFiguresR3_wraf119

SupplementalTable1_ISMER3_wraf119

SupplementalTable2_ISMER3_wraf119

SupplementalTable3_ISMER3_wraf119

SupplementalTable4_ISMER3_wraf119

SupplementalTable5_ISMER3_wraf119

Supplemental_video_1_TM7x_twitching_motility_wraf119

## Data Availability

All unique strains and raw data generated in this study are available upon request but will require completion of a materials transfer agreement. Accession numbers for relevant genomic datasets are listed in Tables S2 and S5.
