# Supplementary material for: Saccharibacteria deploy two distinct type IV pili, driving episymbiosis, host competition, and twitching motility
Source: ISME J. 2025 Jun 9;19(1):wraf119. doi: 10.1093/ismejo/wraf119 (PMC12206443; doi:10.1093/ismejo/wraf119)
Supplement: MergedSupplementalFiguresR3_wraf119 [file mergedsupplementalfiguresr3_wraf119.pdf]

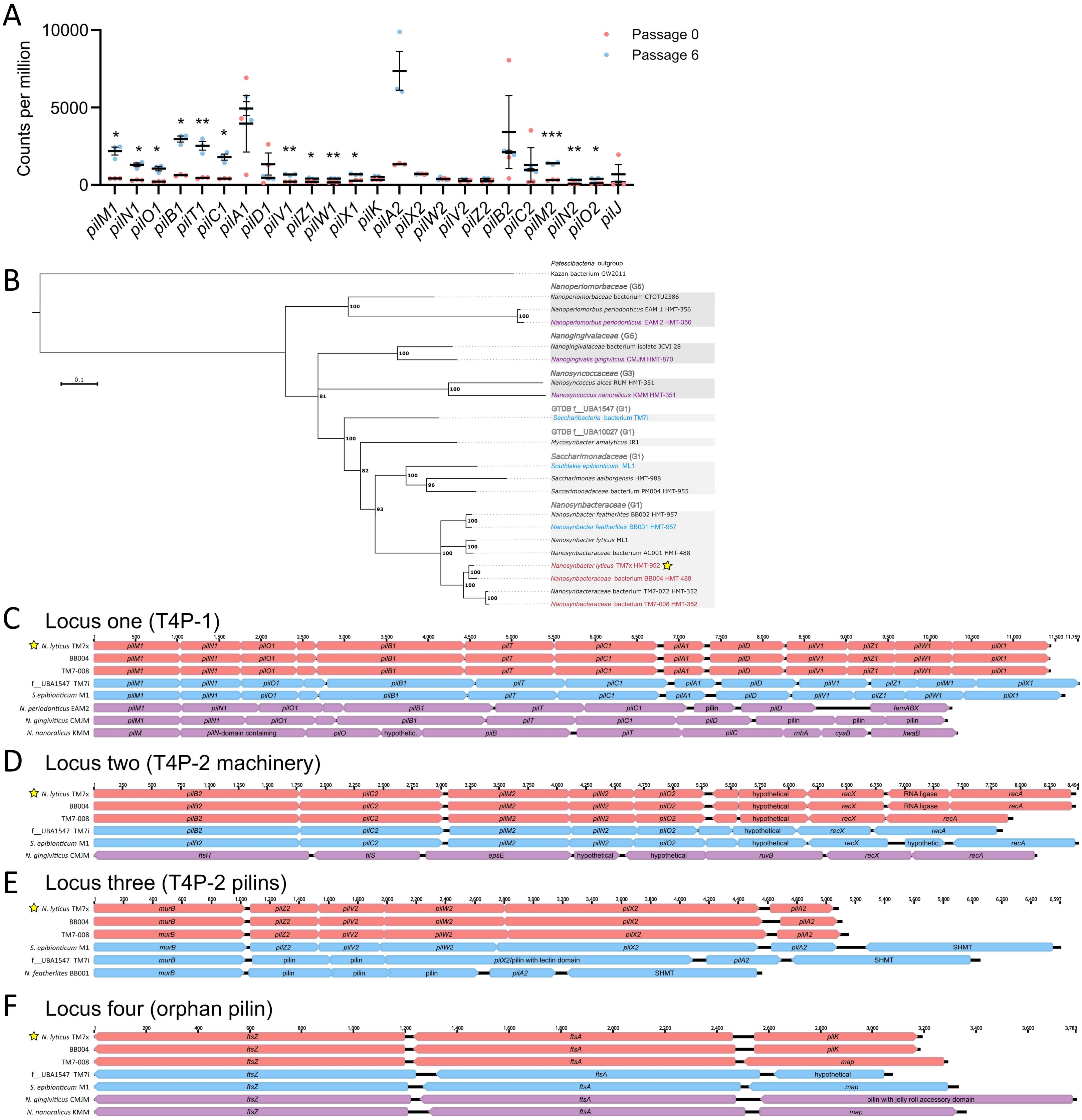

A
Passage 0
Passage 6
Counts per million
10000
5000
0
pilM1 pilN1 pilO1 pilB1 pilT1 pilC1 pilA1 pilD1 pilV1 pilZ1 pilW1 pilX1 pilK pilA2 pilX2 pilW2 pilV2 pilZ2 pilB2 pilC2 pilM2 pilN2 pilO2 pilJ
B
Patescibacteria outgroup
Kazan bacterium GW2011
Nanoperiomorbaceae (G5)
Nanoperiomorbaceae bacterium CTOTU2386
Nanoperiomorbus periodonticus EAM 1 HMT-356
Nanoperiomorbus periodonticus EAM 2 HMT-356
Nanogingivalaceae (G6)
Nanogingivalaceae bacterium isolate JCVI 28
Nanogingivalis gingivitcus CMJM HMT-870
Nanosyncoccaceae (G3)
Nanosyncoccus alces RUM HMT-351
Nanosyncoccus nanoralicus KMM HMT-351
GTDB f__UBA1547 (G1)
Saccharibacteria bacterium TM7i
GTDB f__UBA10027 (G1)
Mycosynbacter amalyticus JR1
Saccharimonadaceae (G1)
Southlakia epibionticum ML1
Saccharimonas aalborgensis HMT-988
Saccarimonadaceae bacterium PM004 HMT-955
Nanosynbacteraceae (G1)
Nanosynbacter featherlites BB002 HMT-957
Nanosynbacter featherlites BB001 HMT-957
Nanosynbacter lyticus ML1
Nanosynbacteraceae bacterium AC001 HMT-488
Nanosynbacter lyticus TM7x HMT-952
Nanosynbacteraceae bacterium BB004 HMT-488
Nanosynbacteraceae bacterium TM7-072 HMT-352
Nanosynbacteraceae bacterium TM7-008 HMT-352
0.1
C
Locus one (T4P-1)
N. lyticus TM7x
BB004
TM7-008
f__UBA1547 TM7i
S.epibionticum M1
N. periodonticus EAM2
N. gingivitcus CMJM
N. nanoralicus KMM
pilM1 pilN1 pilO1 pilB1 pilT pilC1 pilA1 pilD pilV1 pilZ1 pilW1 pilX1
pilin femABX
pilM pilN-domain containing pilO hypothetic. pilB pilT pilC rnhA cyaB kwaB
D
Locus two (T4P-2 machinery)
N. lyticus TM7x
BB004
TM7-008
f__UBA1547 TM7i
S. epibionticum M1
N. gingivitcus CMJM
pilB2 pilC2 pilM2 pilN2 pilO2 hypothetical recX RNA ligase recA
hypothetic.
ftsH tilS epsE hypothetical ruvB
E
Locus three (T4P-2 pilins)
N. lyticus TM7x
BB004
TM7-008
S. epibionticum M1
f__UBA1547 TM7i
N. featherlites BB001
murB pilZ2 pilV2 pilW2 pilX2 pilA2 SHMT
pilin pilX2/pilin with lectin domain
F
Locus four (orphan pilin)
N. lyticus TM7x
BB004
TM7-008
f__UBA1547 TM7i
S. epibionticum M1
N. gingivitcus CMJM
N. nanoralicus KMM
ftsZ ftsA pilK map hypothetical
pilin with jelly roll accessory domain

Figure S1. Conservation of T4P from TM7x across the phylum/class *Saccharibacteria*. Transcriptomic analysis of T4P gene expression during TM7x association **(A)** shows that most T4P genes are upregulated after establishment of stabile episymbiosis (passage 6). Transcriptomic data was extracted from GEO Series: GSE196744 (Hendrickson et al 2022). Exceptionally high expression of certain pilins (*pilA1* and *pilA2*) identifies major pilin subunits. **(B)** Maximum likelihood whole genome phylogeny of select *Saccharibacteria*. Human microbial taxon (HMT) IDs provided for strains within eHOMD. Close relatives of TM7x are indicated in red, intermediate relatives are indicated in blue, and distant relatives are indicated in purple. The depicted scale bar indicates 0.1 nucleotide substitutions per site. **(C)** Examination of T4P locus one indicates that T4P-1 is present in all examined sequences, however it has become highly divergent in TM7-KMM. Examination of T4P locus two **(D)** and three **(E)** indicate strong conservation of T4P-2 amongst all G1 *Saccharibacteria*, and complete absence outside of G1. **(F)** The orphan pilin at locus 4 is specific to TM7x and BB004, however the distantly related TM7-CMJM encoded a different minor pilin at this site. * = P value ≤ 0.05, ** = P value ≤ 0.01, *** = P value ≤ 0.001, **** = P value ≤ 0.0001.

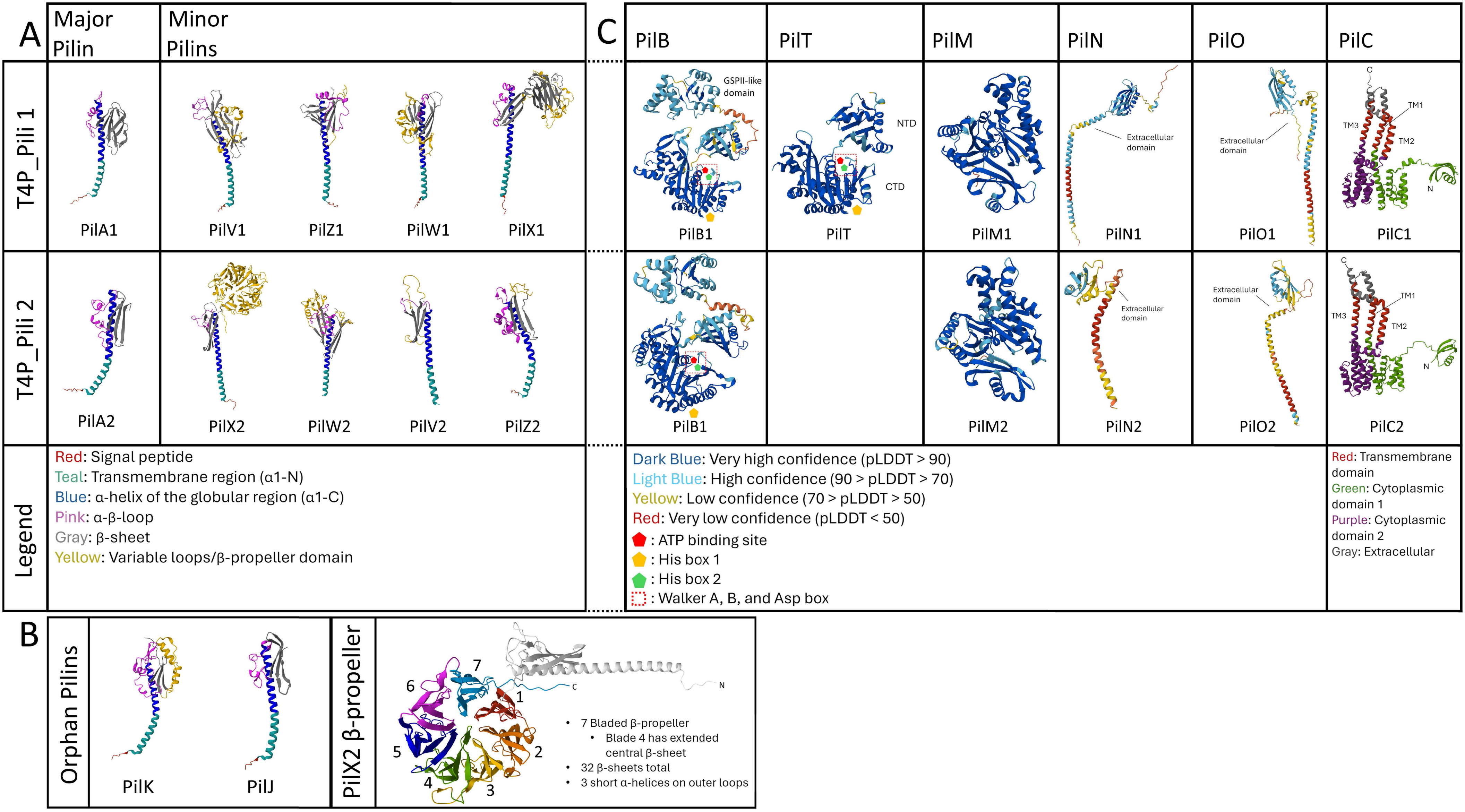
A
Major Pilin
Minor Pilins
T4P_Pili 1
PilA1
PilV1
PilZ1
PilW1
PilX1
T4P_Pili 2
PilA2
PilX2
PilW2
PilV2
PilZ2
Legend
Red: Signal peptide
Teal: Transmembrane region (α1-N)
Blue: α-helix of the globular region (α1-C)
Pink: α-β-loop
Gray: β-sheet
Yellow: Variable loops/β-propeller domain
B
Orphan Pilins
PilK
PilJ
PilX2 β-propeller
1
2
3
4
5
6
7
C
N
• 7 Bladed β-propeller
• Blade 4 has extended central β-sheet
• 32 β-sheets total
• 3 short α-helices on outer loops
C
PilB
PilT
PilM
PilN
PilO
PilC
GSPII-like domain
PilB1
NTD
CTD
PilT
PilM1
Extracellular domain
PilN1
Extracellular domain
PilO1
C
TM1
TM2
TM3
N
PilC1
PilB1
PilM2
Extracellular domain
PilN2
Extracellular domain
PilO2
C
TM1
TM2
TM3
N
PilC2
Dark Blue: Very high confidence (pLDDT > 90)
Light Blue: High confidence (90 > pLDDT > 70)
Yellow: Low confidence (70 > pLDDT > 50)
Red: Very low confidence (pLDDT < 50)
: ATP binding site
: His box 1
: His box 2
: Walker A, B, and Asp box
Red: Transmembrane domain
Green: Cytoplasmic domain 1
Purple: Cytoplasmic domain 2
Gray: Extracellular

Figure S2. AlphaFold2 structural models of T4P proteins from TM7x. Structural models of T4P pilins **(A-B)** and assembly machinery **(C)**, comparing the essential T4P-1 (row one) and the non-essential T4P-2 (row two). T4P-2 has no associated retraction ATPase/PilT. Legends indicate either the model confidence level reported by AlphaFold2 (PilBTMNO) or specific functional structures (PilC and pilins). Special attention is given to the orphan pilins which cannot be confidently assigned to either T4P system as well as the unique PilX2 structure **(B)**.

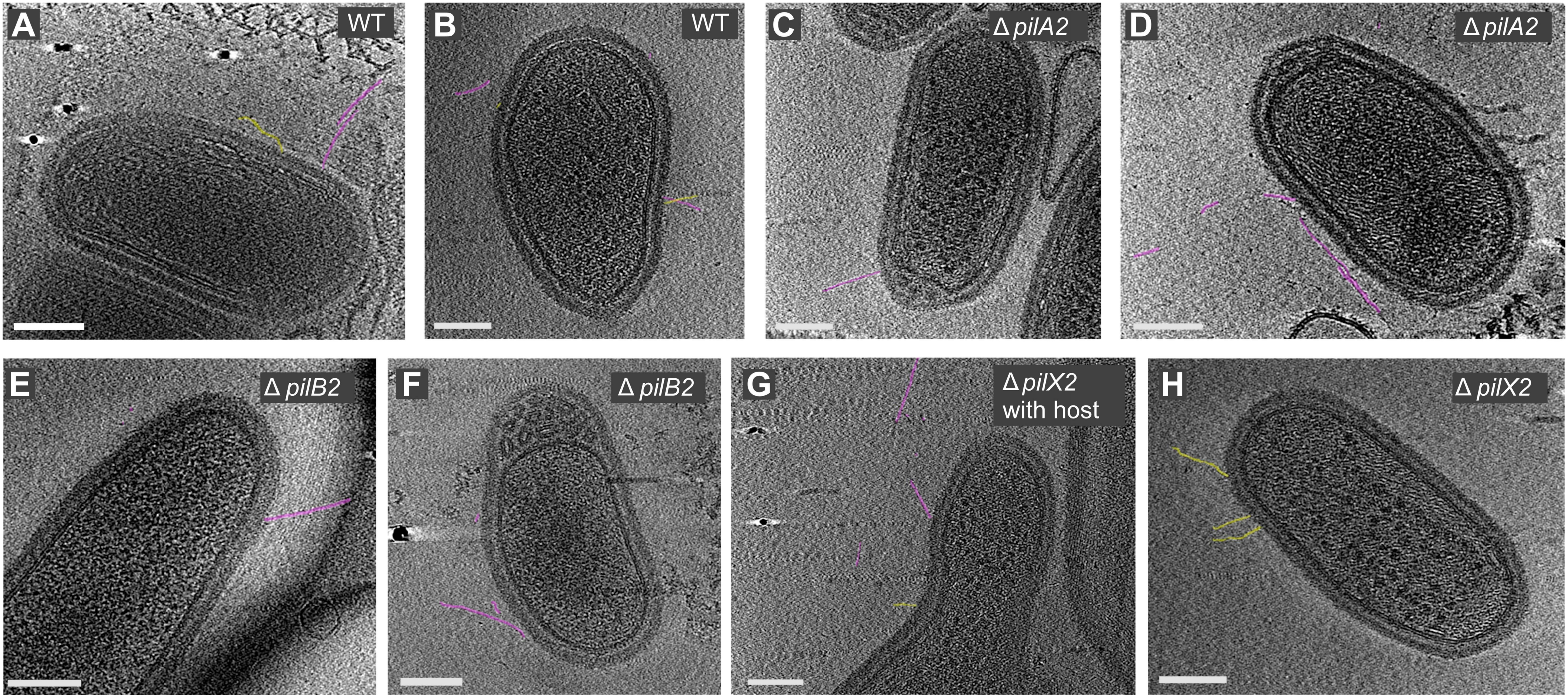
A
WT
B
WT
C
Δ *pilA2*
D
Δ *pilA2*
E
Δ *pilB2*
F
Δ *pilB2*
G
Δ *pilX2*
with host
H
Δ *pilX2*

Figure S3. Expanded Cryo-ET images. Additional electron tomography images of *N. lyticus* TM7x wildtype **(A-B),** TM7xΔ*pilA2* **(C-D)**, TM7xΔ*pilB2* **(E-F)**, and TM7xΔ*pilX2* **(G-H)**. All scale bars are 100 nm. Select pili filaments are highlighted to indicate either thin pili (yellow; diameter ≈1.8 nm) or thick pili (purple; diameter ≈3.2 nm). Thin filaments are only detectable in wildtype cells and TM7xΔ*pilB2*, thick filaments are seen in all treatments. All images show planktonic cells except panel G which shows a cell near, but not in close contact with, a host bacterium (host on right edge).

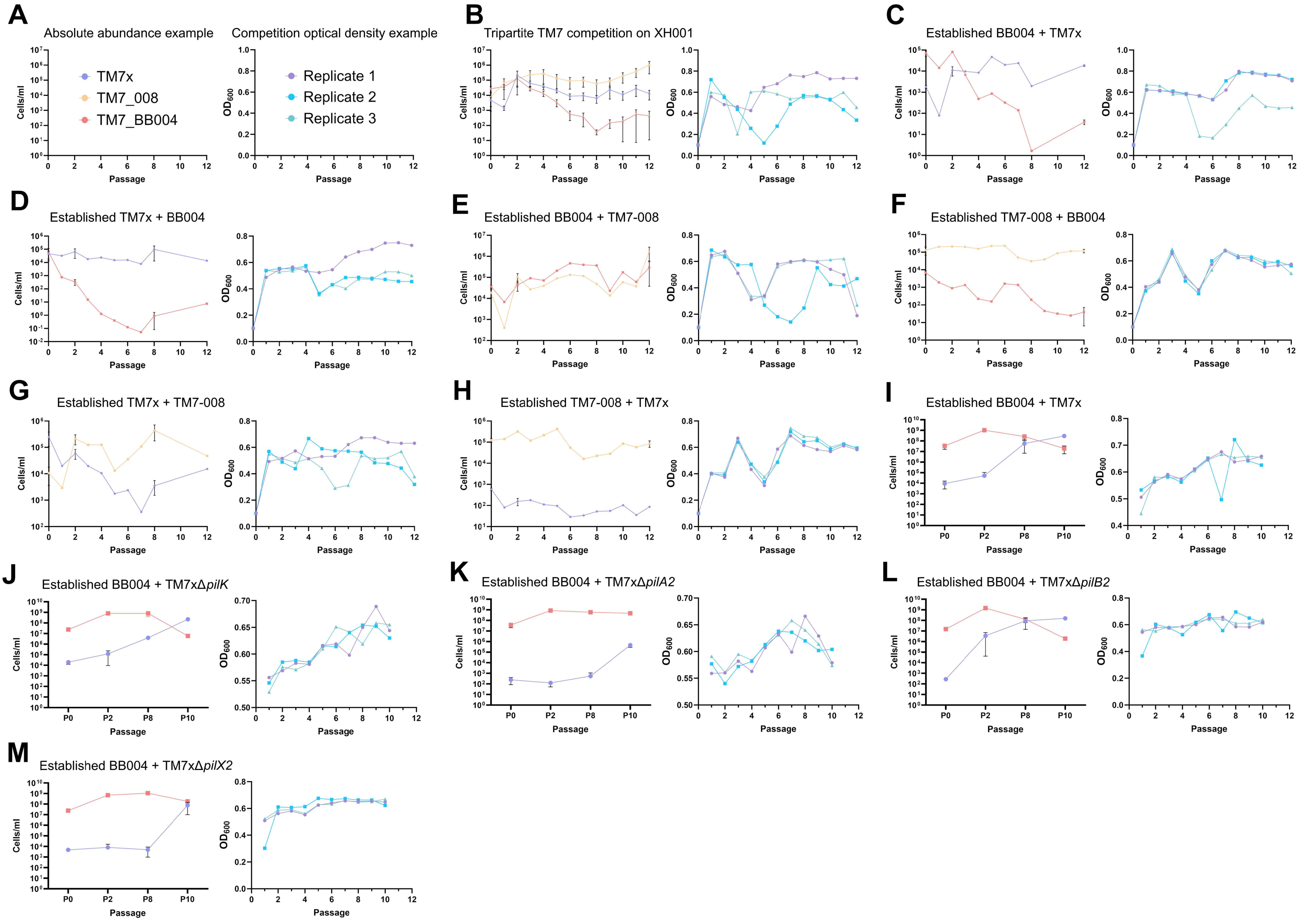
A
Absolute abundance example
Competition optical density example
TM7x
TM7_008
TM7_BB004
Replicate 1
Replicate 2
Replicate 3
B
Tripartite TM7 competition on XH001
C
Established BB004 + TM7x
D
Established TM7x + BB004
E
Established BB004 + TM7-008
F
Established TM7-008 + BB004
G
Established TM7x + TM7-008
H
Established TM7-008 + TM7x
I
Established BB004 + TM7x
J
Established BB004 + TM7xΔpilK
K
Established BB004 + TM7xΔpilA2
L
Established BB004 + TM7xΔpilB2
M
Established BB004 + TM7xΔpilX2
Cells/ml
OD600
Passage

Figure S4. Absolute abundance and OD_{600} data from all competition experiments. Paired qPCR enumeration of TM7x, TM7-008, and BB004 and co-culture optical density values for all competition experiments performed, with universal key in panel **A**. Tripartite competition **(B)** indicates a clear and repeatable outcome where BB004 declines, TM7x persists, and TM7-008 grows to dominance. This is borne out by pairwise combinations of TM7x with BB004 **(C)** which indicate that TM7x has higher competitive fitness on this host. Pairwise combinations of BB004 with TM7-008 **(D)** and TM7x with TM7-008 **(E)** similarly support the ranked competitive fitness observed in the tripartite competition. To examine potential priority effects, each competition was performed twice, each time starting with a different competitor in established co-culture with the host. Competition of BB004 with wildtype TM7x **(F)**, TM7xΔ*pilK* **(G)**, TM7xΔ*pilA2* **(H)**, TM7xΔ*pilB2* **(I)**, and TM7xΔ*pilX2* **(J)** shows that *pilA2* and *pilX2* mutants have decreased competitive fitness.

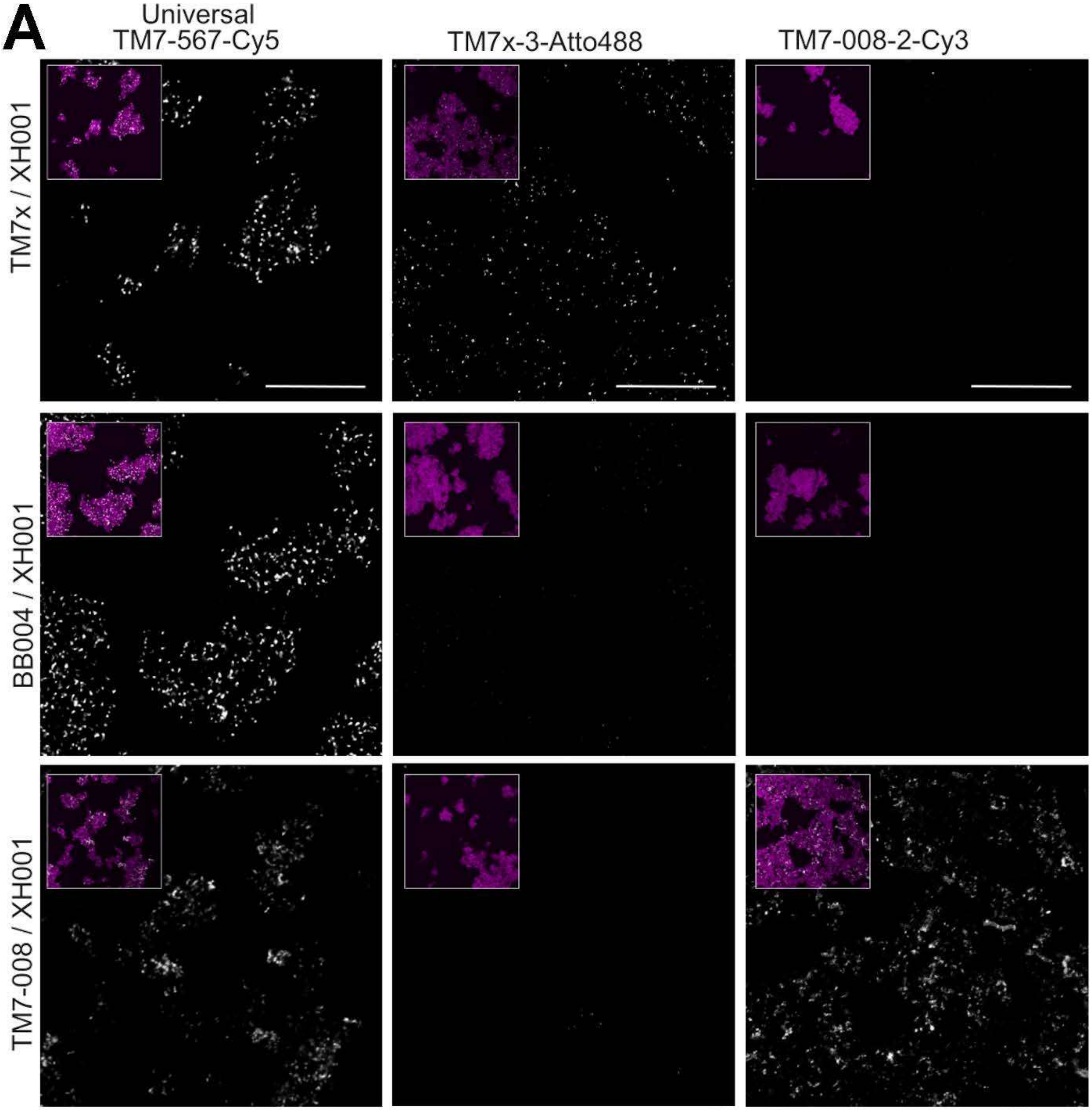

Sup Fig. Scale bars are 20 um.

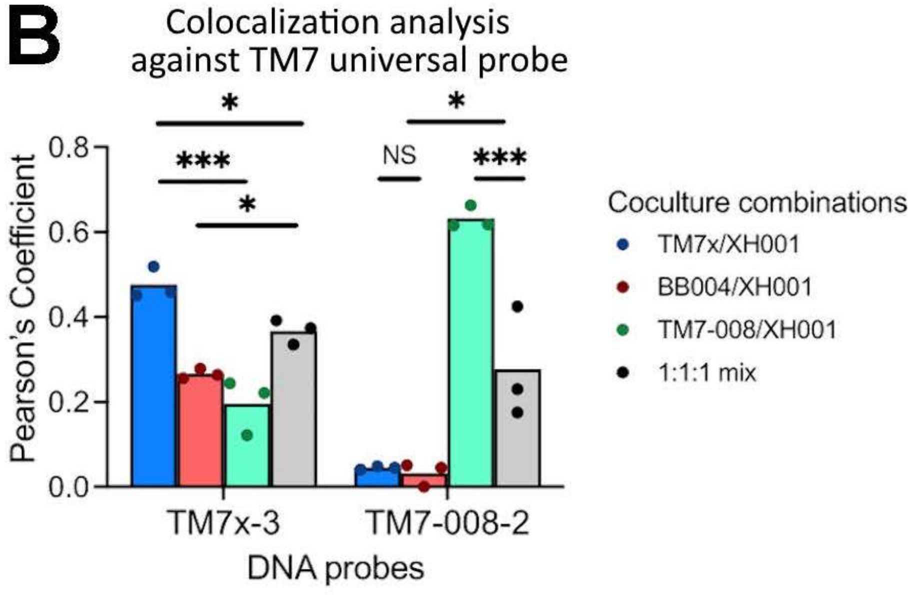

Figure S5. Optimization of strain-specific *Saccharibacteria* FISH probes. To ascertain the specificity of 16S rRNA FISH probes, each probe was conjugated to a distinct fluorophore and used to stain fixed monoxenic co-cultures of *S. odontolytica* containing TM7x, BB004, and TM7-008 **(A)**. To quantify these specificities, cultures were stained with both a strain-specific probe and a universal *Saccharibacteria* probe (TM7-567), then colocalization of both probes, as approximated via the Pearson's coefficient, was used to quantify the binding potential of each probe for each strain **(B)**. Due to the similarity of 16S rRNA sequences, no tested probes were highly specific for BB004, however TM7x-3 displayed some specificity for TM7x, and TM7-008-2 displayed a high level of specificity for TM7-008. * = P value ≤ 0.05, ** = P value ≤ 0.01, *** = P value ≤ 0.001, **** = P value ≤ 0.0001.

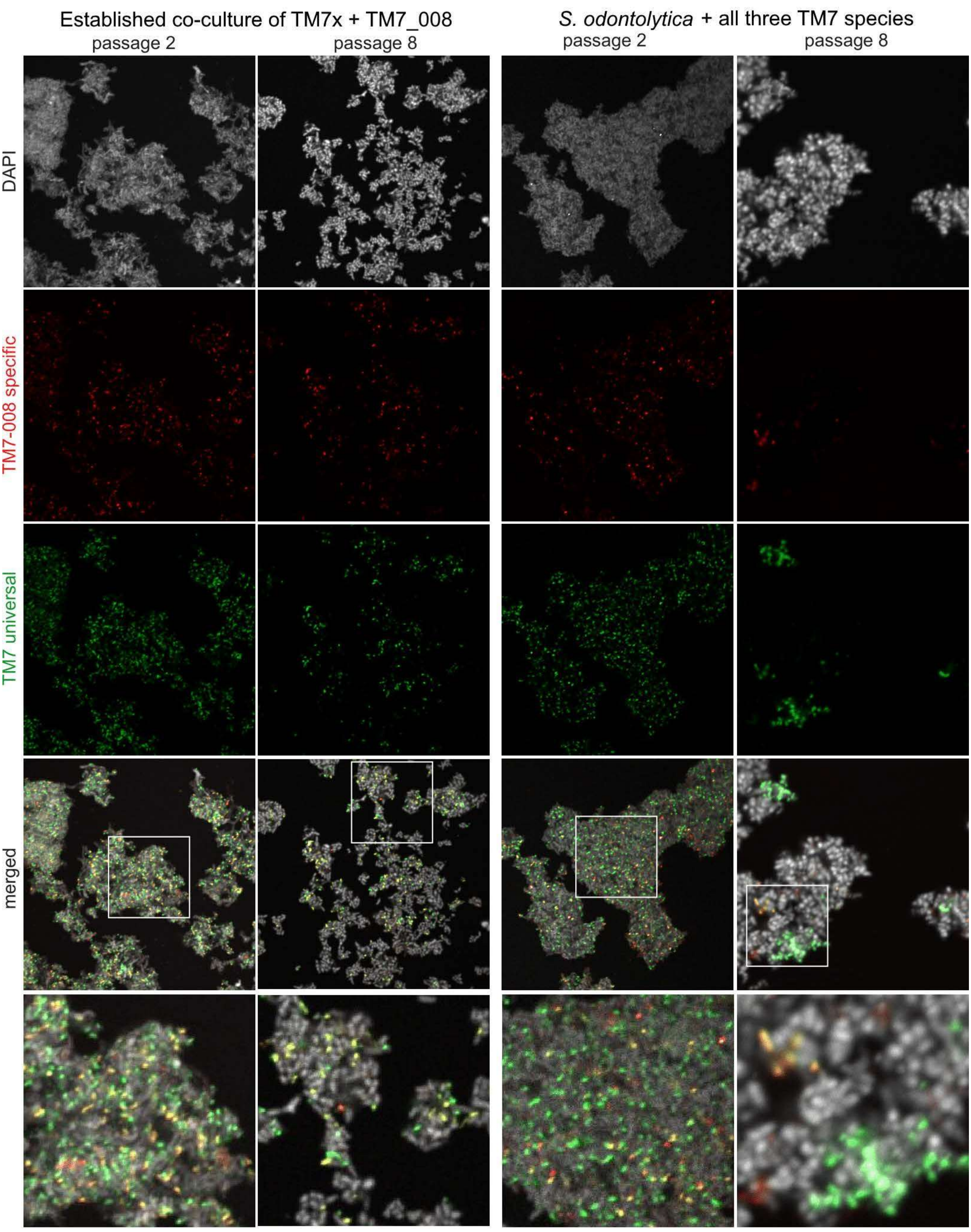
Established co-culture of TM7x + TM7_008
passage 2
passage 8
S. odontolytica + all three TM7 species
passage 2
passage 8
DAPI
TM7-008 specific
TM7 universal
merged

Figure S6. Spatial visualization of *Saccharibacteria* competition cultures. To visualize the fine scale arrangement of competition between *Saccharibacteria* strains for a single bacterial host, fixed co-cultures were stained with DAPI (host cells shown in white), TM7-008-2 probes (TM7-008 specific shown in red), and TM7-567 probes (universal TM7 shown in green). Two distinct competition conditions, established TM7x infected with TM7-008 and tripartite infection, were visualized at two different times, passage two and passage eight post infection. At passage two, competitors were evenly distributed throughout the clumped biomass of *Actinobacteria* with TM7-008 appearing as red or yellow puncti while all other *Saccharibacteria* appear green. Passage eight cultures show more organized arrangement of episymbionts, particularly in the tripartite co-cultures, where distinct strain level clusters are visible amongst the mass of bacterial host cells.
